# Hospital-based retrospective analysis of 224 surgical cases of lung hydatid cyst from southern Iran

**DOI:** 10.1186/s13019-023-02327-w

**Published:** 2023-07-03

**Authors:** Reza Shahriarirad, Amirhossein Erfani, Kamyar Ebrahimi, Mohammad Rastegarian, Mehrdad Eskandarisani, Bizhan Ziaian, Bahador Sarkari

**Affiliations:** 1 Student Research Committee, Shiraz University of Medical Sciences, Shiraz, Iran; 2 Thoracic and Vascular Surgery Research Center, Shiraz University of Medical Sciences, Shiraz, Iran; 3 Department of Surgery, Shiraz University of Medical Sciences, Shiraz, Iran; 4 Basic Sciences in Infectious Diseases Research Center, Shiraz University of Medical Science, Shiraz, Iran; 5 Department of Parasitology and Mycology, School of Medicine, Shiraz University of Medical Sciences, Shiraz, Iran

**Keywords:** Hydatid cyst, Lung, Hospital record, Southern Iran, Surgery, Pulmonary

## Abstract

**Background:**

The lungs are considered the second-most frequent location for hydatid cyst in human. The current retrospective hospital-based study aimed to assess the epidemiological data, clinical presentation, and treatment outcomes of lung hydatid cyst in patients who underwent surgery for this disease in Fars province, southern Iran.

**Methods:**

In this retrospective study, hospital records of 224 pulmonary hydatid cyst patients were assessed in two main university-affiliated hospitals in Fars Province, southern Iran. Clinical features of patients, epidemiological data, cyst features, surgical interventions, and treatment outcomes were reviewed and analyzed.

**Results:**

A total of 224 hydatid cyst cases of the lung were reviewed. Male patients accounted for the majority of cases (60.4%). The average age of the patients was 31.13 (± 19.6), ranging from 2 to 94 years old. Of the 224 patients, 145 (75.9%) cases had only one single cyst and mostly 110 (53.9%) located in the right lung. Also, 6 (2.9%) cases had cysts in both lungs. The lower lobe of the lungs was the most common location of the hydatid cyst. The average size of lung hydatid cyst was 7.37 cm (SD = 3.86; rang: 2–24) while for the cyst areas was 42.87cm^2^ (SD = 52.76; range: 2–488). Regarding the surgical method, 86 (38.6%) cases were operated by lung resection surgery while 137 (61.4%) cases had lung preserving one. The chief complaints of the patients were cough (55.4%) and dyspnea (32.6%). Relapse was documented in 25 (11.16%) of cases.

**Conclusions:**

Lung hydatid cyst is a common infection in southern Iran. Lung preserving surgery is the method of choice for the management of hydatid cyst. Relapse, which was not uncommon in our study, is a challenging feature of hydatid cyst management.

## Background

Cystic echinococcosis (CE) or hydatid cyst, caused by the larval stage of *Echinococcus granulosus*, is a zoonotic helminthic disease that imposes considerable economic losses and is a major public health problem in many countries around the world. CE is endemic in the entire Mediterranean zone, including all countries from the Middle East [1]. Humans become infected through ingestion of food contaminated with parasite eggs, which are excreted in the feces of the dogs. The eggs hatch in the human's gut and the embryo (oncosphere) penetrates the wall of the intestine and is passed through the bloodstream till they reach the vital organs such as the lungs, liver, or brain, where they become hydatid cysts. When an *E. granulosus* oncosphere becomes trapped in an arterial capillary of the lung, the larva develops silently during few years and transforms into a hydatid cyst [2]. Although most of the *E. granulosus* embryos are trapped in the liver, smaller embryos may pass over the hepatic sinusoids and, through the right heart, settle in the lungs. Transdiaphragmatic spreading due to transdiaphragmatic rupture of liver hydatid cyst is considered as the second way of lung infection. The later approach may explain the simultaneous involvement of the liver and lungs with a hydatid cyst [3]. Pulmonary hydatid cyst remains a challenging problem for general and thoracic surgeons in any CE-endemic countries.

Clinical presentation of lung hydatid cysts depends on whether the cyst is ruptured or intact. In the majority of cases, asymptomatic or intact hydatid cysts are detected incidentally during chest X-ray; nevertheless, large cysts may cause symptoms in patients due to the compression of organs adjacent to the lesion. Cough is the main symptom in the majority of pulmonary hydatid cysts cases. In a study by Darwish et al. on 206 patients with pulmonary hydatid cyst, the cough was reported in 54% of cases [4]. In another study on 445 lung hydatid cyst patients, the cough was the chief complaint (79%) of the adults and was much more common (93%) in the children [5]. Breathlessness, chest pain, hemoptysis, and expectoration, are reported as the other symptoms of pulmonary hydatid cysts.

Large pulmonary hydatid cysts can result in complications and increased morbidity [6]. As lungs are softer in consistency than the liver, pulmonary hydatid cysts grow faster than the liver cysts. Moreover, hydatid cysts in children grow at a faster rate and become larger than the adults, owing to their more elastic lung tissues [7, 8].

Hydatid cysts are usually detected as multiple circumscribed oval or solitary masses in radiological imaging [6]. Pneumothorax, suppuration, rupture, and secondary infection are considered as pulmonary hydatid cysts complications [6, 9–15]. Serological diagnosis may help in the diagnosis of pulmonary hydatid cyst [16–18].

The optimal choice for treating lung hydatid cyst of any size is considered to be surgery. Regarding giant pulmonary hydatid cysts, some surgeons prefer pneumonectomy or lobectomy [6, 19] while others suggest the lung preserving surgical methods, regardless of the cyst size [20–22].

Hydatid cyst is a major health problem in Iran where about 1% of all surgeries in hospitals are due to this relatively neglected disease [23–29]. The current hospital-based retrospective study aimed to assess the demographical features, clinical presentation, treatment, and outcomes of lung hydatid cyst in patients who underwent surgery in two main hospitals in Fars province, southern Iran.

## Methods

### Study area and data collection

**Fig. 1:**
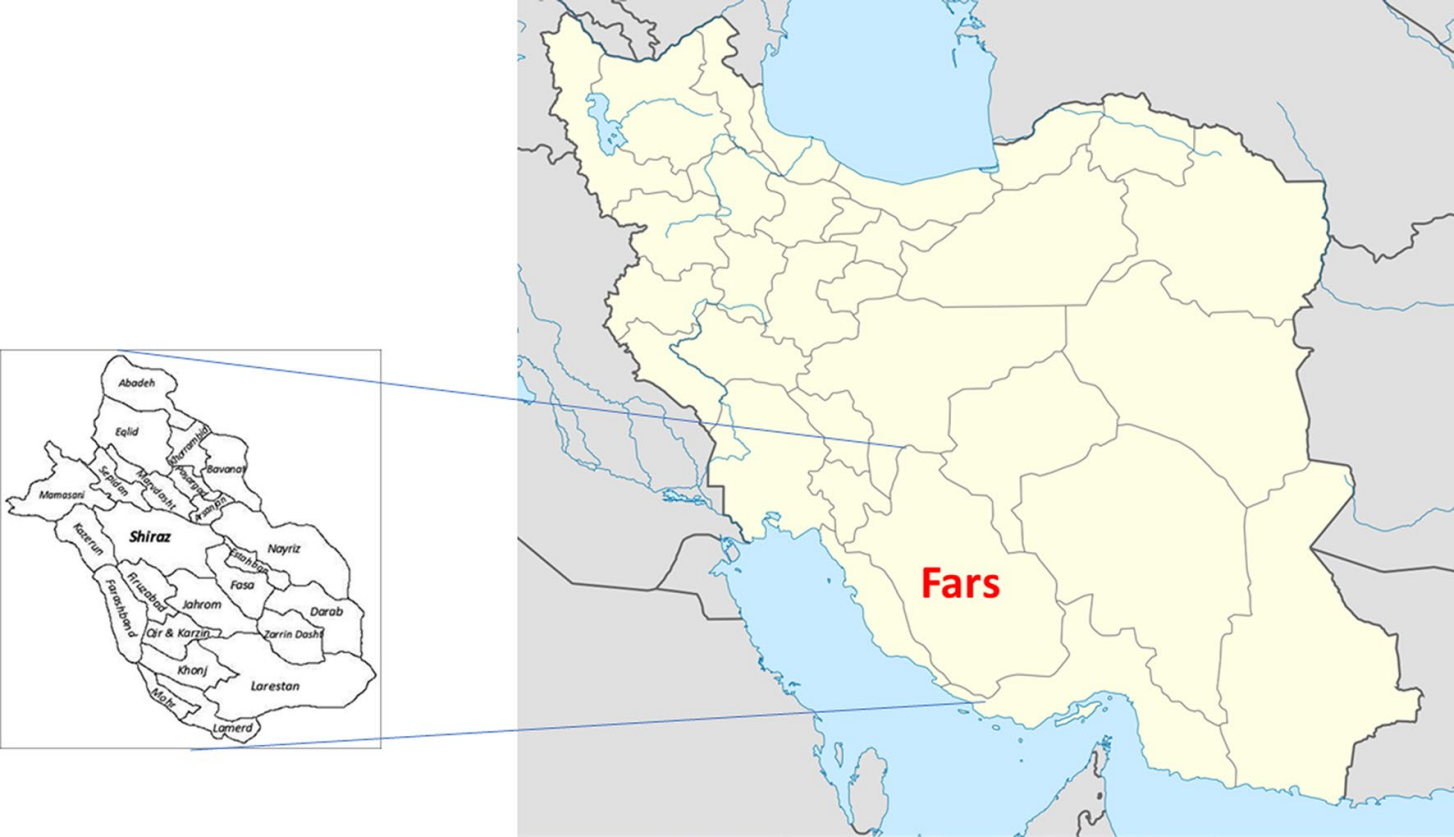
Map of the study area, Fars province, southern Iran

This study was conducted in Shiraz, the capital of Fars Province in southern Iran (Fig. 1). The population of Shiraz is 1,869,001(the fifth-most-populous city in Iran). Hospital records of patients, who underwent surgery due to pulmonary hydatid cysts during a 15-years period (2004–2018) in two main university-affiliated and referral hospitals (Shahid Faghihi and Namazi) in Fars Province in southern Iran, were reviewed. The preliminary diagnosis of hydatid cyst in all cases has been well-defined by postoperative pathological findings. Thus, operation with the diagnosis of CE and post-operative confirmation by a pathological approach were the inclusion criteria for any pulmonary hydatid cases, while reviewing the records.

The hospital medical records of all patients were carefully reviewed by members of our team which included both medical students and physicians. The demographic and clinical data existing in the patients’ records including age, sex, place of residence, cyst features (number, location, size, multicystic, multiloculated, daughter cyst, septation, rupture, inflammation based on radiology report, superimposed infection, and calcification), sign and symptoms, duration of hospitalization, treatment modalities, surgical procedures, the outcome of treatment, and history of relapse and other hydatid cyst relevant data were recorded. In our study, for measuring the area of the cyst, the square centimeters of the area of cysts was calculated using the area of the oval/circle formula; which was the smallest radius (half of the smallest diameter) multiplied by largest radius (half of the largest diameter) by Pi (3.14) which was used for all imaging modalities.

### Statistical analysis

Collected data were analyzed, using SPSS software, version 28 for Windows (SPSS Inc.). The data were checked regarding their normal distribution with the Komarov-Shapiro test, and parametric data were reported as mean and standard deviation (SD), while non-parametric data were reported as median and quartiles. Demographic and clinical data of patients were tabulated. Statistical analyses were performed, using the Chi-square test for independence or categorical dependent variables. Significance level was set at *P* < 0.05.

## Results

**Fig. 2:**
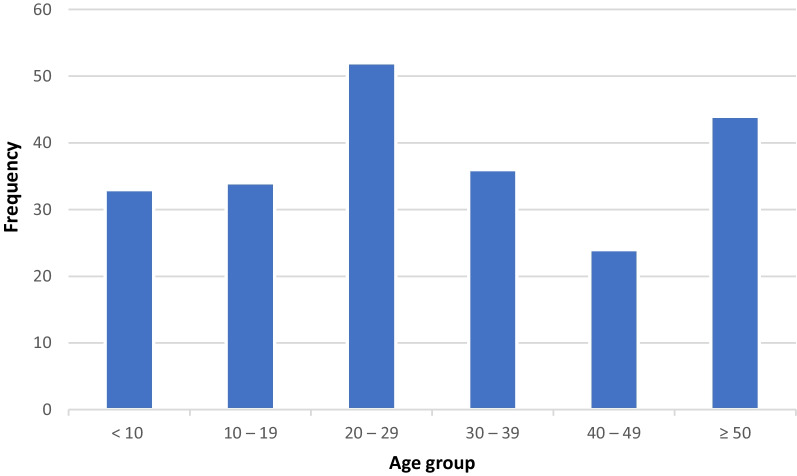
Frequency of lung hydatid cyst in 224 cases from Southern Iran based on age groups

A total of 224 hydatid cyst cases of the lung were reviewed in this retrospective study. The majority of cases (60.4%) were male. The patients’ age ranged from 2 to 94 years old with a mean age of 31.13 (± 19.6) years. Figure 2 shows the frequency of hydatid cyst in the lung among various age groups. Also, the demographic and clinical features of patients with pulmonary hydatid cysts in the current study are demonstrated in Table 1.

**Table 1:** Demographic and clinical features of lung hydatid cyst and its distribution among different age groups in 224 cases from Southern Iran

Variable	Frequency (%)	Age terms				*P* value
		Children (< 16) *n* = *63*	Young adult (17 – 30) *n* = *61*	Middle age adult (31- 45) *n* = *46*	Old adult (> 45) *n* = *53*	
*Gender*						0.724
Male	134 (60.4)	43 (68.3)	33 (55)	29 (63)	28 (53.8)	
Female	88 (39.6)	20 (31.7)	27 (45)	17 (37)	24 (46.2)	
*Duration of hospital stay (days)*						
Median [IQR]	10 [9]	9 [7]	12 [11]	10 [10]	11 [8]	0.199
< 6	33 (15)	10 (16.1)	8 (13.1)	8 (18.2)	7 (13.2)	0.490
6–10	85 (38.6)	26 (41.9)	19 (31.1)	19 (43.2)	21 (39.6)	
11–15	44 (20)	11 (17.7)	13 (21.3)	7 (15.9)	13 (24.5)	
16–20	32 (14.5)	8 (129)	12 (19.7)	5 (11.4)	7 (13.2)	
21–30	21 (9.5)	4 (6.5)	7 (11.5)	5 (11.4)	5 (9.4)	
> 30	5 (2.3)	3 (4.8)	2 (3.3)	–	–	
*Location of hydatid cyst in lung*						
Right	110 (53.9)	27 (49.1)	31 (54.4)	26 (61.9)	26 (52)	0.561
Left	88 (43.1)	28 (50.9)	22 (38.6)	15 (35.7)	23 (46)	
Bilateral	6 (2.9)	–	4 (7)	1 (2.4)	1 (2)	
*Number of cysts*						
Median [IQR]	1 [1]	1 [0]	1 [1]	1 [1]	1 [1]	0.328
1	145 (75.9)	42 (84)	40 (71.4)	29 (72.5)	33 (75)	0.374
2	34 (17.8)	8 (16)	9 (16.1)	9 (22.5)	8 (18.2)	
≥ 3	12 (6.3)	–	7 (12.5)	2 (5)	3 (6.8)	
*Largest Size of cyst diameter (cm)*						
Median [IQR]	7 [5]	7 [4]	7 [5]	6.5 [4]	6 [4]	0.660
≤ 5	57 (38.5)	16 (37.2)	20 (46.5)	4 (9.3)	3 (7)	
6–10	67 (45.3)	12 (31.6)	16 (42.1)	8 (21.1)	2 (5.3)	
11–15	19 (12.8)	12 (37.5)	17 (53.1)	3 (9.4)	0 (0)	
> 15	5 (3.4)	17 (48.6)	14 (40)	4 (11.4)	0 (0)	
*Area of the cyst (cm*^*2*^*)*						
Median [IQR]	27.48 [35]	27.48 [35]	27.87 [64]	28.26 [31]	19.63 [30]	0.731
< 26	68 (48.2)	18 (42.9)	17 (50)	13 (43.3)	20 (57.1)	0.094
26–50	38 (27)	14 (33.3)	4 (11.8)	11 (36.7)	9 (25.7)	
51–75	8 (5.7)	2 (4.8)	3 (8.8)	3 (10)	–	
76–100	16 (11.3)	6 (14.3)	4 (11.8)	2 (6.7)	4 (11.4)	
> 100	11 (7.8)	2 (4.8)	6 (17.6)	1 (3.3)	2 (5.7)	
*Cyst features*						
Ruptured	19 (8.5)	6 (9.5)	5 (9.8)	1 (2.2)	6 (11.3)	0.511
Calcified	12 (5.4)	3 (4.8)	2 (3.3)	4 (8.7)	3 (5.7)	0.279
Infected	16 (7.1)	–	3 (4.9)	6 (13)	4 (7.5)	**0.008**
Isolated	1 (0.4)	–	–	1 (2.2)		0.460
Multioculated	3 (1.3)	–	–	1 (2.2)	2 (3.8)	0.257
Fibrosis	2 (0.9)	1 (1.6)	1 (1.6)	–	–	1.000
With ectocyte	1 (0.4)	–	–	1 (2.2)	–	0.201
Inflammation	1 (0.4)	–	–	1 (2.2)	–	0.201
Necrosis	2 (0.9)	–	1 (1.6)	–	1 (1.9)	0.707
*Coinfection*						
Liver	43 (19.2)	17 (27)	7 (11.5)	6 (13)	13 (24.5)	**0.011**
Spleen	2 (0.9)	–	1 (1.6)	1 (2.2)	–	0.460
Heart	1 (0.4)	–	–	1 (2.2)	–	0.575
Mediastinum	1 (0.4)	–	–	–	1 (1.9)	–
Subdiaphragmatic area	2 (0.9)	–	–	–	2 (3.8)	0.094
Common bile duct	1 (0.4)	–	1 (1.6)	–	–	–
Pancreas	1 (0.4)	–	–	–	1 (1.9)	–
*Surgery method*						
Lung resection	86 (38.6)	21 (33.3)	25 (41)	19 (41.3)	21 (39.6)	0.605
Lung preserving	137 (61.4)	42 (66.7)	36 (59)	27 (58.7)	32 (60.4)	

Bold values demonstrate significant association

IQR: Interquartile Range

The frequency of cysts based on the involving lobe of the lung is shown in Fig. 2.

Among the 224 reviewed records, the incidence of hydatid cyst relapse was documented in 25 (11.16%) cases in which 21 (7.6%) patients had 2 times, 3 (1%) patients had 3 times, and 1 patient (0.4%) had the experience of 4 times relapse.

**Fig. 3:**
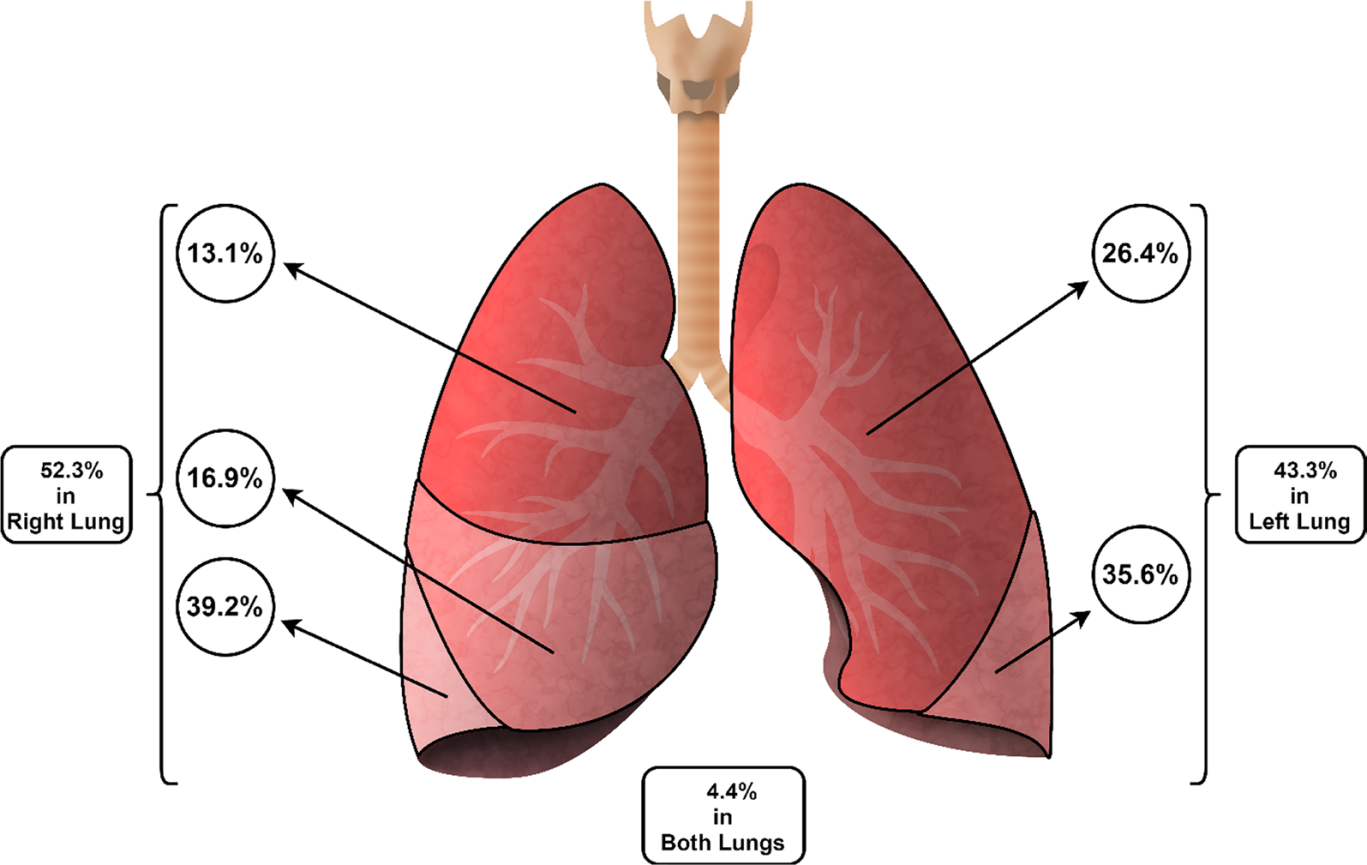
Distribution of lung hydatid cysts based on location in the lung lobe (the present image is ours and was drawn by KE, the fourth author of the article)

Among the total cases of lung hydatid cyst, 145 (75.9%) cases had only one single cyst, and mostly 110 (53.9%) were located in the right lung. Also, 6 (2.9%) cases had cysts in both lungs. The right lower lobe was the most common location of the cyst in the right lung while the left lower lobe was the most common location of the cyst in the left lung (Table 2). The frequency of cysts based on the involving lobe of the lung is shown in Fig. 3.

**Table 2:** Location of hydatid cyst and method of surgery in 224 pulmonary hydatid cyst cases in southern Iran

Location in Lung	Frequency (% of organ/total)	Surgery (% within an organ)	
		Lung resection surgery	Lung preserving surgery
Right			
Right upper lobe	21 (12.8)	6 (28.6)	15 (71.4)
Right middle lobe	24 (17.3)	8 (33.3)	16 (66.7)
Right lower lobe	58 (38.9)	31 (53.4)	27 (46.6)
Left			
Left upper lobe	42 (31.1)	17 (40.5)	25 (59.5)
Left lower lobe	46 (34.3)	24 (52.2)	22 (47.8)

The size of lung hydatid cyst varied between 2 and 24 cm (median = 7; Q1–Q3: 5–10; mean = 7.37, SD = 3.86) and the cyst areas varied between 2 till 488 cm^2^ (median: 27.48; Q1–Q3: 15.70–50.24; mean 42.87, SD = 52.76). Coinfection was recorded in 16 cases of hydatid cyst, 3 (1.3%) of them was due to fungal infections.

Considering the lung hydatid cyst imaging, 126 out of 127 (99.2%) CT scans reported lung hydatid cyst. Also, 42 out of 51 (82.4%) sonographies reported positive in lung hydatid cyst, 25 out of 27 (11.2%) chest X-ray reported positive hydatid cyst of the lung.

Regarding the surgical method, 86 (38.6%) cases were operated by lung resection surgery while 137 (61.4%) cases underwent lung preserving (cystectomy and cavity management) surgery. Furthermore, in 90 (40.2%) of cases evacuation of hydatid cyst, 24 (10.7%) excision, 26 (11.6%) resection, 18 (8%) laparotomy, 10 (4.5%) drain insertion, 30 (13.4%) unroofing of hydatid cyst have been performed (Table 2).

Considering the chief complaints of the patients on admission, 124 (55.4%) had cough, 73 (32.6%) had dyspnea, 58 (25.9%) had related pain to location of cyst, 58 (29.4%) had fever, 23 (10.3%) had hemoptysis, 17 (8.7%) had chills, 8 (3.6%) had nausea/ vomiting, 4 had weight loss (1.8%), and 3 (1.3%) had anorexia.

The most frequent medications administered for patients were third generation cephalosporins (Ceftriaxone), which was prescribed for 203 (72.8%) cases. Furthermore, Lincosamides (Clindamycin) were prescribed for 213 (76.3%) patients, antihelmetic (Albendazole) for 170 (60.9%) patients, Mebendazol in 3 (1.1%), Metronidzaole for 59 (21.1%), Fluroquinolones (Ciprofloxacin) for 75 (26.9%), first generation cephalosporins (Cephalothin, Cefazolin, and Cephalexin) for 27 (9.7%) cases and carbapenems (Meropenem) in 6 (2.2%) patient. Aminoglycosides in 7 (2.5%), Macrolides in 2 (0.7%), and Glycopeptides in 10 (3.6%) cases were administered.

Based on the laboratory findings of the patients, considering the white blood cell count (WBC), 113 of cases had leukocytosis, 9 had eosinophilia, 31 had neutrophilia, 36 had lymphopenia, and 6 cases had monocytopenia.

Based on the type of surgery and the relapse of hydatid cyst among cases, 16 (53.3%) were reported among lung preserving surgery methods while 14 (46.7%) were among lung resection surgery. Also, regarding the post-op hospital stay duration, there was no significant difference among the two groups (lung preserving 12.25 ± 8.1, lung resection 12.58 ± 8; *P* value = 0.749).

Based on the rehospitalization records of our patients, in which male gender (OR = 2.12, CI95%: 1.16–3.86; *P* = 0.013) and also cyst in the both lungs (*P* = 0.014) had a significant association with relapse.

## Discussion

Lung is known as the second most frequent site of hydatid cyst. Surgery retains the dominant role in the treatment of hydatid disease of the lung. Evacuation of lung cysts and elimination of the endocyst accompanied by maintenance of the residual cavity and opening of air licking airways is the purpose of surgery in the treatment of pulmonary hydatid cysts. For lung hydatid cysts, lung protection strategies are typically favored in children and adult patients [30–32].

In 1946, Ugon et al. [33] and Barrett in 1947 [34, 35] were the first to incorporate cyst enucleation, followed by purse-string sutures (capitonnage) to obliterate the remaining cavity. Today, this is the most commonly used pulmonary hydatid cyst treatment technique [36]. However, it is better to puncture the cyst and aspirate the contents of the cyst for large cysts with considerable stress [36–38], which significantly promotes the removal of the cyst [39]. The lung, and particularly the cyst-containing lobe, should be released from any adhesion to the chest wall before opening the cyst. Spillage of the contents of the cyst into the thoracic cavity is normal after needle aspiration [30, 40, 41]. Prophylactic procedures include covering the underlying tissues with pads immersed in 15 percent saline solution and to irrigate the cyst cavity with a scolicidal agent (e. g. 10–15% saline solution) [30, 42].

Lung preserving surgery (cystectomy and cavity management) is a more common treatment for hydatid cyst and this has been the main approach for the management of the lung hydatid cyst. In a surgery report of hydatid cyst in pediatric patients, Cangir et al. recommended avoiding lung resection surgery in children [43], which further studies also approved this mindset for adult patients [8, 44]. In our study, 61.4% of the cases undergone lung preserving surgery for the hydatid cyst with no noticeable difference regarding the relapse and duration of hospital stay, compared to those who underwent lung resection. Rapture is the most frequent complication of pulmonary hydatid cyst which may lead to significant clinical consequences. In the current study, rapture of hydatid cyst was noted in 19 (8.9%) of cases which is somewhat less than those reported in other studies [45].

Regarding gender, in line with other studies [37, 46, 47] we have noted a predominance of the disease in males (60.4%) in comparison with the females (39.6%). Such association has been seen in the hydatid cyst of other organs as well [25, 27].

It has been reported that the hydatid cyst of the lung is more common than the liver in the children [5, 7, 8]. Moreover, concomitant lung and liver involvement are more common in adults than in children. In a retrospective study by Kanat et al. the medical records of 145 patients with hydatid disease were reviewed. The concomitant hepatic cyst was reported in 33% of children whereas this rate was 79% for adults [48]. Another study by Khatonaki et al. reported only 9% cases of CE in Jahrom city in Fars province, to be localized in the lung [49]. In our study, about a third of the lung hydatid cyst cases (31.1%) were in children which indicate a high prevalence of lung involvement in this group.

With their special clinical characteristics, cysts with a large diameter are called giant hydatid cysts. In the pediatric group, giant cysts are more prominent because of the greater elasticity of the lungs in children, which enables cyst expansion [38, 50, 51]. In the current study cyst with larger size were more common in young adults and children in comparison with middle age and old adults, although the differences were not statistically significant.

The hydatid cyst has been reported to be able to grow anywhere in the lung, however it resides on the right side more frequently than on the left side and has a preference for the lower lobe. While the presence of hydatid cyst in the left lobe was more prominent in some reports [52], several studies have shown pulmonary hydatid cyst to be more commonly located in the right and lower lobes [50, 53–55]. In Kocer et al. [56] study, no difference was noted between the involvement rates of the two lobes. In our study, the disease was more common in the right lobe, especially in the right lower lobe of the lungs.

In the current study, most patients (75.9%) had only a single cyst while 6% of cases had more than three cysts. Aytaç et al. [46] reported that 72% of hydatid cyst patients in Turkey had single cysts, 15% had multiple unilateral cysts and 13% had multiple bilateral cysts. In Aubert and Viard's [57] study, 82.6% of patients had single cysts, 10% had several unilateral cysts, and 7.4% had multiple bilateral cysts.

In few studies, the rate of coexisting hepatic and pulmonary involvement has been reported to be between 7 and 18% [52, 54], while in a large series, hepatic and pulmonary involvement rates were less than 10% [30]. In the current study, hepatic and pulmonary involvement was seen in 19.2% of cases while simultaneous involvement of lung and spleen or subdiaphragmatic was seen in only 2% of cases.

A 10 years (1999–2009) retrospective study on human hydatid cysts cases in two main hospitals of Tehran, central Iran, revealed 177 cases, 162 with liver, and 15 cases of lung involvement [58]. Cough was reported in 40% of the cases while hemoptysis was recorded in 26.67% of the cases. In Arinc et al. study, 138 patients with pulmonary hydatid cyst were evaluated where chest pain and cough (44.9% and 37.6%) were reported as the most frequent symptoms of the disease [19]. Analysis of 106 patients with a lung hydatid cyst in Kolkata revealed that 14.15% of the patients were asymptomatic. Cough was the most common symptom (73.58%) followed by chest pain (54.72%) [59].

Clarification is also required regarding the necessity of postoperative regular albendazole therapy. According a report by Akbulut et al. [60] regarding pulmonary hydatic cyst, adjuvant albendazole medication is not advised in cases without the probability of remnant disease or relapse, such as after surgery for solitary cysts, uncomplicated cysts, or patients who underwent severe surgery [61]. The use of neoadjuvant albendazole in lung hydatic cyst is debatable. According to certain research, neoadjuvant albendazole treatment is advised for cysts with a small diameter, many cysts, and substantial intraoperative spread risks. According to numerous studies, neoadjuvant therapy should be avoided because there is a significant chance of subsequent rupture [11].

In Bagheri et al. study, [9] among the 1024 patients with lung hydatid cyst, the mean age of patients was 30.6 years, and the male/female ratio was reported to be 1.09. Cough was reported as the most common symptom (55.1%). Almost all, except 1%, of patients, were symptomatic. The right lung was involved in 53.8% of cases, while bilateral involvement was reported in 6.2% of cases. Removing the cyst membrane, without resecting the pericyst, and closing the airways was the most common (67.2%) surgical management [9]. In a review of 763 cases of lung hydatid cyst in Iraq, 37% were asymptomatic with the incidental diagnosis on routine chest X-ray [62].

Among the limitations of our study, we can include the retrospective nature and incomplete hospital record documentations of these cases. Also, we only included surgical cases of hydatid cyst, and our report cannot be representative of the total number of pulmonary hydatid cysts in our region. Furthermore, our study lacks long-term follow-up and the possibility of recurrence. Further studies in this regard are warranted to increase our understanding of the exact burden of this disease, along with the most effective measures for management and treatment.

## Conclusion

Lung hydatid cyst is a common infection in southern Iran. The disease is common in children and male constitutes the majority of cases. The right lower lobe is the most common location of the cyst. Lung preserving surgery is the method of choice for the treatment of hydatid cyst. Relapse, which was not uncommon in our study, is a challenging feature of pulmonary hydatid cyst management.

## Data Availability

The datasets used and/or analyzed during the current study are available from the corresponding author on request.

## Abbreviation

CE: Cystic echinococcosis
